# Predicted impact of mosquito-stage transmission-blocking vaccines using an ensemble of microsimulations

**DOI:** 10.1186/1475-2875-9-S2-O11

**Published:** 2010-10-20

**Authors:** Aurelio Di Pasquale, Nicolas Maire, Thomas Smith

**Affiliations:** 1 Department of Epidemiology and Public Health, Swiss Tropical and Public Health Institute, 4051 Basel, Switzerland; 2 University of Basel, P.O. Box, CH-4003 Basel, Switzerland

## 

Dynamic models of infectious diseases have played an important role in the rational planning of control programs for a long time. We have developed a comprehensive microsimulation approach to investigate the potential of many of the currently possible and future interventions against *P. falciparum* malaria. The outputs of the stochastic individual-based simulations are predictions of the epidemiological impact and comparative cost-effectiveness of conceivable control measures, alone or in combination.

The analysis of such simulation studies is challenging because they can produce very large numbers of outputs. Usually a large number of scenarios need to be investigated, especially if the focus is on how different control intervention act in combination. In addition, uncertainty analysis requires the re-running of the model with a number of differing model formulations or parameters.

We are developing a web-based platform to be able to efficiently design, run and analyze simulation experiments. Here we present an overview of the architecture of the platform, including the underlying database design, the workflow of running simulations, and the tools for analyzing predictions. The platform will greatly improve the accessibility of the model predictions to end-users from a range of disciplines, such as program managers or policy makers. We also discuss as a case study the results from a simulation experiment aimed a predicting the potential value of deploying a mosquito-stage transmission-blocking vaccine (MSTBV) against *P. falciparum* malaria. These results suggest how a MSTBV can best be combined with other control measures to achieve elimination, considering factors like the initial level of transmission, the proportion of the population covered by the intervention, and the key properties of the vaccine.

